# Threats and harassment of public health officials in the United States during the COVID-19 pandemic: insights from the adaptive leadership framework

**DOI:** 10.3389/ijph.2026.1609739

**Published:** 2026-09-01

**Authors:** Jeffrey Glenn, Mirabella A. Keogh, Katelyn N. Aldridge

**Affiliations:** Brigham Young University Department of Public Health, Provo, UT, United States

**Keywords:** adaptive leadership, COVID-19, public health, public health workforce, workplace violence

## Abstract

**Objectives:**

Many public health officials faced threats and harassment while doing their jobs during the coronavirus disease 2019 (COVID-19) pandemic. The Adaptive Leadership Framework (ALF) serves as a useful lens through which the dangers of exercising leadership in a crisis situation can be analyzed and understood.

**Methods:**

This study employed a systematic qualitative media content analysis to examine U.S. newspaper coverage of public health officials during the COVID-19 pandemic. We identified articles using Newspaper Source Plus and Newswires (EBSCO) databases and then screened and coded them using a deductive approach guided by the ALF. A total of 50 articles were analyzed using directed thematic analysis.

**Results:**

Media coverage revealed widespread and multifaceted resistance to public health leadership during the pandemic. Opposition originated from both the public (e.g., harassment, threats, and protests) and authority figures (e.g., firing). Despite these challenges, public health officials demonstrated adaptive leadership behaviors, particularly maintaining stability and building strong partnerships.

**Conclusion:**

Threats and harassment reflected broader institutional and political pressures, underscoring the importance of institutional protection and leadership preparation for future public health crises.

## Introduction

The coronavirus disease 2019 (COVID-19) pandemic was an unprecedented global public health emergency that presented public officials with daunting leadership challenges. Between February 2020 and December 2021, the pandemic led to over 18 million deaths worldwide and over 1 million deaths in the United States [1]. The crisis proved politically divisive as public health experts and elected officials were required to make rapid, high-stakes decisions amid scientific uncertainty, evolving epidemiological evidence, and intense public scrutiny. Public officials experienced different forms of harassment and opposition throughout the pandemic, including death threats and job termination [2]. In retrospect, the leadership challenges these officials faced offer insights into the dangers of exercising leadership. The Adaptive Leadership Framework (ALF) serves as a useful lens through which to examine these challenges and learn more about “staying alive” in leadership when the stakes are high [3].

### Leadership during COVID-19

Research emphasizes that public health leadership played a key role in increasing public trust and achieving good health outcomes during the COVID-19 pandemic [4, 5]. Multiple studies have identified evidence-based decision-making as a foundational leadership practice. Leaders who relied on scientific expertise and public health guidance were more successful in establishing credibility and sustaining public adherence to mitigation measures such as masking, physical distancing, and vaccinations [4, 6]. Additionally, transparent and consistent communication fostered trust and ensured compliance with mitigation efforts, especially when leaders were required to implement restrictive policies [4, 7, 8]. Adding to these insights, research suggests that leaders who prioritized empathy, stewardship, and emotional support were better able to sustain morale among healthcare workers and maintain organizational stability during prolonged uncertainty [6].

Some studies report high rates of physical violence, verbal abuse, and cyber harassment experienced by public health officials in their leadership roles during the COVID-19 pandemic [9–11]. One survey revealed that approximately one-third of U.S. public health workers reported at least one form of workplace violence, including stigma, job-related threats, and bullying or harassment [10]. Evidence suggests that poor workplace conditions and harassment towards public health officials led to high rates of poor mental health, burnout, quitting, or contention between public officials [12–15]. Survey-based research on local health department leaders found that over 57% reported experiencing verbal abuse, threats of violence, or lawsuits, with 88% indicating that such harassment negatively affected their mental health and 66% reporting staff shortages that compounded organizational strain [16]. These findings underscore how workplace violence and harassment during COVID-19 were both widespread and consequential, extending beyond isolated incidents and becoming embedded within broader institutional and political contexts [11]. While many studies have analyzed leadership during COVID-19, the ALF offers unique insights into the nature of the leadership challenges and the harassment faced by public health officials.

### Adaptive leadership framework

The ALF was developed by Ronald Heifetz, Marty Linsky, and colleagues at the Harvard Kennedy School as a tool for understanding and exercising leadership when faced with adaptive problems [3, 17, 18]. Heifetz defines adaptive problems, in contrast to technical problems, as those driven by underlying conflicts in values, beliefs, and loyalties where the exact nature of the problems is hard to define and the solutions are unknown [17]. These problems are challenging because addressing them requires people to change their ways and build something new. Across international and domestic contexts, COVID-19 has been framed not merely as a technical challenge of leadership but as a complex and adaptive problem requiring emotional intelligence, continuous learning, and flexibility [6, 7]. The pandemic presented the world with innumerable instances of adaptive problems, among the most pressing being how to overcome political and cultural differences to unite against the spreading virus. While the lack of definitive scientific consensus at the outset only intensified the adaptive nature of the work needed to make progress against the virus, the inherent tension between scientific experts and elected officials made the work fundamentally adaptive in nature [19].

The ALF distinguishes between leadership exercised by individuals who are in positions of authority and leadership without authority, recognizing that leadership can come from those with or without authority but that the opportunities and constraints between the two types of leadership differ [17]. Leadership can therefore be considered a practice or activity rather than a job someone is assigned.

Another key feature of the ALF is that it highlights the inherent danger of exercising adaptive leadership since, by definition, addressing these problems requires challenging people’s core values and beliefs [17]. Doing so generates opposition or resistance on behalf of those being pushed to change their ways which, in the case of COVID-19, regularly surfaced in the form of harassment towards officials doing their best to make public decisions in the face of uncertainty. Heifetz and Linsky highlight historical examples of individuals being literally assassinated for exercising adaptive leadership, and they point out that assassination can also happen figuratively as those exercising leadership are ignored, marginalized, or otherwise neutralized for representing controversial perspectives that another faction in the social system wants to silence [3]. Figurative assassination of public health officials in the form of threats and harassment was common during the pandemic.

When faced with the uncomfortable reality that they may be part of the problem, stakeholders often engaged in work avoidance, which Heifetz et al. define as conscious or unconscious patterns of behavior that distract people’s attention from the difficult work required of meeting an adaptive challenge. Work avoidance may include behaviors such as focusing only on the technical parts of a problem, engaging in interpersonal conflict, or, as evident in the many examples of harassment of public officials during the pandemic, the scapegoating and attacking of authority figures. Because of the strong opposition to and danger faced by those exercising adaptive leadership on adaptive issues, the ALF emphasizes the central role of purpose as a driving motivation for being willing to exercise leadership.

In contrast to traditional hero models of leadership in which the leader is the person with authority who claims to have all the answers, the ALF recognizes the importance of giving the work back to others by mobilizing them to take responsibility for solving problems. The ALF identifies and recommends productive behaviors that can help those exercising adaptive leadership to give the work back. These include listening with curiosity and compassion in heated situations. There are times when exercising leadership requires holding steady by remaining steadfast in the face of opposition rather than overreacting or attempting to respond to every criticism. Those exercising leadership must be willing to accept responsibility for their difficult decisions and for the possibility that they may play a role in perpetuating the problem. Partnering is another important act of leadership in which those exercising leadership collaborate with others who share their points of view across or within organizations. Support from these partners can be critical to identifying the underlying issues and moving forward with a plan of action.

As noted above, many studies have explored the importance of strong leadership during the COVID-19 pandemic, and others have sought to document the harassment faced by public officials during this period. Ours is the first to apply the ALF as an analytical tool to specific stories of harassment to identify insights into exercising leadership on adaptive problems in the face of serious opposition. While the ALF emphasizes the importance of and opportunities for leadership without authority, the focus of this study is on leadership from public officials in positions of authority during the COVID-19 pandemic.

## Methods

### Study design

This study employed a systematic qualitative media content analysis to examine U.S. newspaper coverage of public health officials during the COVID-19 pandemic. The purpose was 1) to identify and categorize incidents of harassment (defined as all forms of opposition from the public and from elected officials, including threats, doxxing, stalking, verbal abuse, firing, etc.) and 2) to examine how leadership behaviors described in media accounts aligned with constructs from the ALF. Since newspaper coverage does not account for all instances of harassment towards public health workers, the results should be viewed as an analysis of media portrayals of such incidents.

### Data collection

The study focused on U.S.-based newspaper articles published between January 1, 2020, and 31 December 2023, a period encompassing the height and aftermath of the COVID-19 public health response. We conducted a comprehensive literature search using Newspaper Source Plus and Newswires (EBSCO) databases, accessed through the Brigham Young University library. Searches were limited to newspaper publications within the specified date range. As shown in Table 1, the search strategy used Boolean operators and incorporated key terms related to COVID-19, public health leadership, harassment, political pressure, and professional turnover.

**TABLE 1:** Search terms (United States of America, 2020–2023).

• COVID-19 terms (Title) COVID-19 OR coronavirus OR covid OR pandemic OR lockdown • Public health leadership terms (All Text) public health leader* OR public health official* OR health expert* OR elected official* OR epidemiologist* OR health commissioner* OR health officer* OR public health director* OR health secretary* OR chief medical officer* OR health minister* OR health administrator* OR health regulator OR health executive* OR public health administrator* • Harassment and politicization terms (All Text) incivility OR harass* OR anti-vax* OR abuse* OR assault OR violence* OR polarize* OR intimidate* OR threat* OR political pressure OR bullying* OR discriminate* OR politicization • Turnover terms (All Text) resign* OR quit* OR fire* OR terminate* OR dismiss*

**FIGURE 1:**
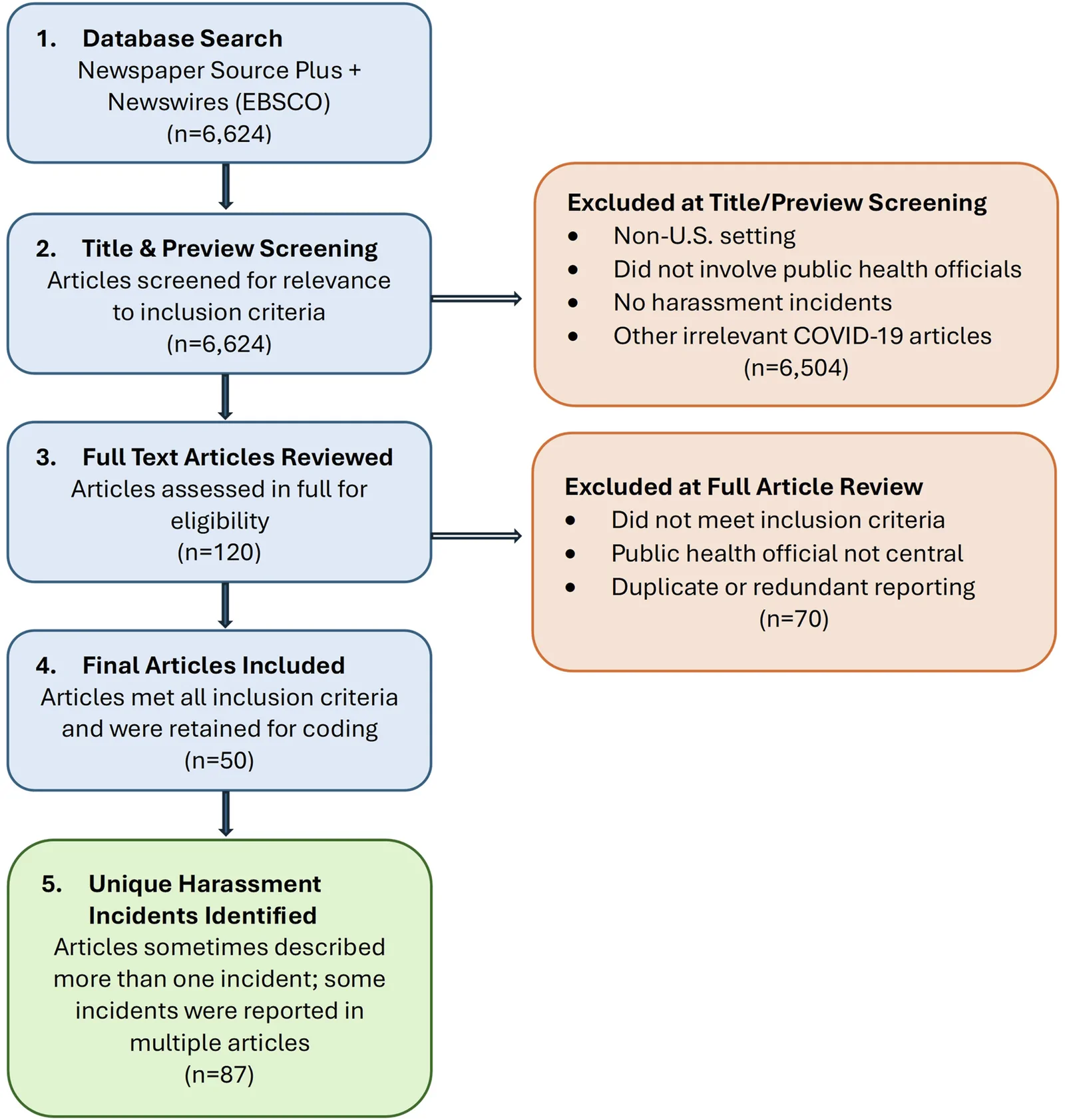
Flow diagram of newspaper article screening (United States of America, 2020-2023).

Figure 1 illustrates the article identification, screening, and selection process. The initial search yielded 6,624 newspaper articles. Three reviewers independently screened article titles and previews (displayed in the EBSCO search results) to determine relevance. Articles were excluded if they 1) did not involve public health officials in the United States (defined as public health officials or other public officials whose responsibilities related to public health) or 2) were unrelated to harassment, threats, political pressure, or professional turnover. Articles with ambiguous relevance were retained for full-text review. This first round of screening resulted in 120 total articles. In the final round of screening, two reviewers conducted a full-text review of all remaining articles, reviewing a subset of 20 articles together to establish consistency in applying inclusion and exclusion criteria. After confirming uniformity, the reviewers divided the remaining articles and conducted independent full-text reviews. Discrepancies were resolved through consensus and bi-weekly meetings, resulting in a final study dataset of 50 articles.

### Data analysis

Once the initial and full-text screening were completed, we developed a theory-informed codebook through an iterative process informed by a review of the ALF, existing literature on public health leadership, and the initial screened articles. We refined and finalized the codebook through team discussion and pilot coding, with definitions clarified to ensure conceptual consistency and analytic rigor. The final codebook characterized different types of harassment and their consequences, and it operationalized key ALF constructs, including holding steady, partnering, giving work back, listening, demonstrating empathy, accepting responsibility, and fighting back. Two reviewers then uploaded and coded the final sample of articles in Dedoose qualitative data analysis software (Version 9.0.107). Each reviewer reviewed and coded half of the articles before jointly reviewing all of the coded articles together to ensure consistency in applying the codebook. Following the article screening and coding, relevant data were extracted and analyzed using a directed thematic analysis focused on identifying recurring patterns related to different forms of harassment and leadership behaviors exhibited by public health officials in the dataset. Frequencies were reported to organize and characterize the relative prominence of themes across coded newspaper articles, consistent with recommendations for the appropriate use of numbers in qualitative research, rather than to estimate prevalence or imply statistical generalizability [20].

## Results

Our final sample consisted of 50 newspaper articles published in the United States between 1st January 2020, and 31st December 2023. Included articles were published throughout the study period (Supplementary File 1), although most appeared in 2020 and 2021, corresponding to the period of greatest public health restrictions and media attention. Selected articles reported on 87 unique incidents of harassment (some articles reported on multiple incidents and some incidents were reported in multiple articles) experienced by public health officials at all levels of government. However, most articles in the sample focused on incidents involving state and local officials. Articles reported instances of harassment from the public as well as harassment directed to public health officials from elected officials in positions of authority. Many articles also described how public health officials responded to harassment, providing opportunities to investigate examples of the ALF in action.

### Harassment

#### Forms of harassment

Public health officials experienced harassment from two directions: from the public (e.g., angry county constituents) and from elected political officials (e.g., state legislators). The type of harassment varied considerably depending on the source. We organized the various categories as represented in Tables 2, 3, providing quotes to illustrate.

**TABLE 2:** Harassment from the public (United States of America, 2020–2023).

Type	Quote
Violent threats (n = 28)	“Deputies advised public health officer tamalee st. James robinson that a man had written a letter asking the sheriff to provide weapons and venue so he could confront her in a duel. St. James robinson said the man, whom she had never met, wanted to prove she “would go to the length of killing someone to carry out the mitigation measures” for COVID-19.” (article 3)
Verbal harassment (n = 15)	“Khan was surrounded by an angry mob, he said, and called the c-word and a brown b-----d. Others mocked his accent.” (article 47)
Threats to family (n = 10)	“Collins and his family were threatened with physical assault and death if collins continued to speak about the need for “mandatory COVID-19 vaccinations.” (Article 32)
Violent acts (n = 8)	“A motorist tried to run him off the road - twice - at more than 70 mph.” (article 3)
Social media (n = 8)	“There’s been many over the course of 8 months, to personal attacks on facebook calling me every name in the book, to calling me and cussing me and saying I’m stupid and I’m incompetent and I don't know what I’m doing, of course the pandemic is fake, and all those type of things.” (article 16)
Protests (n = 6)	“A meeting of the four-county Idaho health district that includes boise was brought to an abrupt end when protesters gathered not only outside the district office but banged on the doors of board members participating remotely from home.” (article 49)
Doxxing (n = 6)	“Public health officials in Colorado were doxed by a GOP leader who posted their names and home addresses in a facebook group along with the message: ‘Take this information and make your own decisions’.” (article 23)
Legal actions (n = 5)	“Local public health officers have been on the front lines in dealing with the disease and now unfortunately they’re being put at the front lines of the litigation involving these, but I’m optimistic that this lawsuit will go nowhere.” (article 9)
Stalking (n = 3)	“Kristina lawson, president of the medical board of California, claimed that she was stalked by a group of anti-vaccination armed extremists, who also used drones to conduct surveillance … ‘to start the day the group parked their rental SUV near the end of my driveway, and then flew a drone over my house’.” (article 21)
Hate mail (n = 1)	“St. James robinson, overwhelmed by hostile facebook posts, hate mail, protests outside her office and the workload of the pandemic, resigned.” (article 3)

* Counts represent coded instances identified in the analyzed newspaper articles and are presented only to orient readers to the relative prominence of themes. They should not be interpreted as estimates of the prevalence of harassment incidents.

**TABLE 3:** Harassment from authority Figures (United States of America, 2020–2023).

Type	Quote
Verbal criticism (n = 13)	“She endured an incredible amount of unfair criticism, including some anti-semitic attacks.” (Article 3)
Firing (n = 11)	“She spent time speaking in national media outlets in rebuttal to a firing she argues was political appeasement for republican lawmakers.” (Article 43)
Blocking hiring (n = 1)	“Evers in a year-end interview said he saw ‘no reason why’ the chamber’s republican majority hadn't acted on the outstanding nominations. ‘They’re doing everything that’s asked of them, and it just boggles my mind how some really quality people are just left hanging like this’.” (article 1)

* Counts represent coded instances identified in the analyzed newspaper articles and are presented only to orient readers to the relative prominence of themes. They should not be interpreted as estimates of the prevalence of harassment incidents.

Our coding revealed that harassment from the public took many forms, including 28 unique instances of violent threats, eight instances of violent acts, 10 examples of threats to family, 15 cases of verbal harassment, six instances of protests, eight examples of harassment over social media, three cases of stalking, one instance of hate mail, five mentions of legal action, and six unique instances of doxxing.

Public health officials also experienced harassment from the top down. This appeared in our sample as harassment by elected officials, including legislators, governors, or, in some cases, the President of the United States. Harassment generally took three forms, including 11 instances of firing, one example where the public health official experienced the blocking of hiring, and 13 instances of verbal criticism.

#### Consequences of harassment

Harassment had serious effects on the mental, physical, and financial state of officials. Our sample revealed three main effects of harassment on public health officials (see Table 4). Firstly, particularly as a result of violent harassment from the public, in 16 unique instances, officials expressed high levels of fear for their personal safety, for the safety of their family, and, in some cases, for the safety of other officials and peers. Burnout was also found, with 12 separate officials reporting feeling severely emotionally, physically, and mentally taxed. There were 28 cases of officials who chose to resign from their positions rather than face the continued risk of harassment.

**TABLE 4:** Consequences of harassment (United States of America, 2020–2023).

Consequence	Quote
Resignation (n = 28)	“Citing the need to protect her children and after receiving another promising job offer as a nurse, elliott resigned last week as director.” (article 16)
Fear (n = 16)	“When I heard that message, I felt cold inside,” she said. ‘It still has an emotional toll. I’m constantly watching over my back.’ (article 3)
Burnout (n = 12)	“She worked 10–12 h a day for 90 days in a row. But she could handle that, she said. She could handle people disobeying expert advice. That on top of the harassment, however, left her with no other choice.” (article 16)

* Counts represent coded instances identified in the analyzed newspaper articles and are presented only to orient readers to the relative prominence of themes. They should not be interpreted as estimates of the prevalence of harassment incidents.

### Leadership behaviors aligned with the adaptive leadership framework

Our coding identified seven leadership behaviors described in the ALF that public health officials exhibited in response to incidents of harassment (see Table 5).

**TABLE 5:** Leadership behaviors aligned with adaptive leadership framework (United States of America, 2020–2023).

Behavior	Quote
Partnering (n = 25)	“The school district asked for a more prudent look at the data. I Was confident that there was an outbreak,” smith said. He ran the data past two epidemiologists at the state health department, who agreed with him, advising that these were epidemiologically linked cases.” (article 43)
Holding steady (n = 22)	“I think some people wanted to stick it out,” she says. “They didn't want to leave in the middle of the COVID crisis.” (article 4)
Accepting responsibility (n = 12)	“We’ve learned a lot of lessons; we definitely need more staff resources and manpower, but there are ways that we could do this work more efficiently and effectively to maximize our impact in the community.” (article 4)
Fighting back (n = 11)	“Republican gov. Ron DeSantis’s administration fired Florida’s former chief coronavirus data officer rebekah jones in May after she said she refused to manipulate data that justified the state’s aggressive reopening plans. She has now created her own COVID-19 tracking site, and claims the state is still hiding important data.” (article 25)
Giving work back (n = 6)	“We have a perspective from each state that is valuable and should be considered as decisions are made,” levine said. “We all need to work together.” (article 30)
Listening (n = 4)	“Several health department volunteers who previously worked with bishai spoke in support of his reinstatement, pointing to his efforts to listen to and mobilize the community.” (article 12)
Demonstrating empathy (n = 4)	“What’s so great about this work, and the experience that our public health AmeriCorps members will have, is that they will be on the ground, talking to residents about their experiences and giving them information,” says romo.” (article 4)

* Counts represent coded instances identified in the analyzed newspaper articles and are presented only to orient readers to the relative prominence of themes. They should not be interpreted as estimates of the prevalence of harassment incidents.

There were 25 instances of partnering, with Kentucky serving as a pertinent example. Kentucky was extremely hard hit by the pandemic due to a lack of resources after the Department of Health experienced severe fiscal constraints dating back to 2008 [21, 22]. The Health Department received a generous grant from AmeriCorps to assist in the department’s COVID efforts, in large part thanks to the partnerships formed by the department*. “For a smaller organization like us to get this kind of grant, we’ve just been thrilled,”* reported Dana Cox Nickles, executive director of the Kentucky Health Departments Association (KHDA). *“We were encouraged to apply by our national association, and we had help writing the grant from state partners who will be helping us with the training. It’s been a joint effort.”* In a different example of partnering, following her resignation from the Kanawha-Charleston Health Department, Dr. Sherri Young indicated that the support she received from her peers was an upside of the job, despite significant harassment. “*You have incredible support from the people you work with,” Young said. “You’re working to save lives, and you’re doing it together.”*

Another leadership behavior exhibited by public health officials was holding steady, with 22 instances. Despite experiencing harassment on all sides, public health officials continued to work and push forward public measures. As Dr. Karen Landers, a health officer with the Alabama public health department, expressed, “*We know viruses will mutate, and SARS-CoV-2 is no different, as we saw before with delta. The core preventive measures haven’t changed. We’re going to continue to apply the same standards that we have for the last 2 years.”* In advising public health officials on how to respond to harassment, Dr. Anthony Fauci, director of the National Institute of Allergy and Infectious Diseases, stated, *“People calling for you to be beheaded, fired, thrown in the fire pit or whatever, that’s just noise. You don't pay attention to that.”*

Accepting responsibility was a leadership behavior found 13 times in the coded articles. For example, in King County, Washington, Mr. Duchin witnessed what few public health officials were lucky enough to experience: widespread public support and praise for his leadership. However, Duchin publicly focused on his administration’s failures: *“We’re seeing the consequences now of a complete and utter failure to ensure we have a full and robust vaccination system,”* he reportedly said. Some public health officials who experienced vicious harassment reported feeling sobered, with public health board members who saw severe backlash in Arizona feeling enthusiastic about their work, with one board member stating, *“You actually now know what you do is important. The decisions you make as an elected official have ramifications.”*

Many public health officials fought back (n = 11), especially when facing harassment from leadership. Following her firing as chief coronavirus data officer in Florida after refusing to cede to Governor Ron DeSantis' demands to falsify data to justify reopening plans, Rebekah Jones created her own COVID-19 tracking site. In Hawaii, state epidemiologist Jennifer Smith acted as a whistleblower, exposing mistreatment of public health officers in the state’s health department. *“People of our community have been confused, frustrated and anxious. I spoke out because the people of Hawaii deserve not only to be as safe and healthy as possible during the pandemic; they deserve to know the facts,”* Smith said.

Public health officials demonstrated the behavior we coded as giving work back in six unique instances. In an interview with a reporter, Governor Mike DeWine of Ohio expressed his frustration that the public is not more routinely involved in discussions surrounding who has responsibility in containing the virus. “*I think our job at this point, all of us, 11.7 million Ohioans, is to do what we can to slow the spread,*” he stated. “*The thing that is going to slow our recovery and could halt it in its tracks is if people are scared and back off … The rebound of the economy is tied at the hips to how well we are doing in fighting the coronavirus. If we start losing that battle, the economy will not recover. That’s what is sometimes missed in this discussion … people*.” In another instance, Dr. Rachel Levine, the director of Pennsylvania’s Department of Health, criticized the Department of Health and Human Services’ inability to give work back to the states, creating severe distribution problems with antigen testing kits. “*We have a perspective from each state that is valuable and should be considered as decisions are made*,” Levine said. “*We all need to work together.”*

Listening and demonstrating empathy were each found four times in our sample. Following the firing of Health Officer David Bishai in Hartford, fellow officers highlighted his *“efforts to listen to and mobilize the community”*. In Hawaii, when questioned on why she continued to work under the harsh conditions, Smith stated her motivation was empathy for the people in need: *“We all wanted to help people, but we didn't have the resources to do it.”*

## Discussion

This study examined how U.S. newspaper coverage portrayed harassment directed at public health officials during the COVID-19 pandemic and how these experiences aligned with the ALF. Our analysis found that harassment was widespread, multifaceted, frequently severe, and commonly involved threats of violence. The willingness of the public to threaten violence against officials and the number of times this occurred raises serious concerns about the safety of public health officials in times of crisis.

Importantly, public health officials faced harassment from the members of the public but also from elected officials. These findings highlight the need to consider workforce protection as a central component of planning for public health crises. Institutional safeguards, legal protections, and mental health support may be necessary to ensure that public health leaders can exercise authority during future crises while avoiding excessive personal risk.

Research suggests that the many cases of harassment during the pandemic were not solely driven by public opposition but were often exacerbated by structural and political factors [2]. For example, in a study that identified more than 1,400 pandemic-related harassment incidents and 222 position departures between March 2020 and January 2021, the mixed-methods analysis further identified structural and political challenges, citing the undermining of public health expertise and intensified social backlash as key contributors to workplace violence against public health officials [2]. Additionally, supporting studies specifically cited the gaps in protective legislation and funding for public health officials as contributors to the distress of public health leaders and areas that need improvement for the future [14].

The application of the ALF provided additional insight into the dynamics of resistance and leadership behaviors observed during the pandemic. In response to opposition, we found many examples of public health officials “holding steady”, continuing to pursue protective policies despite severe harassment received from the bottom up and the top down. Although we saw all these examples in our sample as positive leadership behaviors, holding steady taken to an extreme may take the form of avoidance of necessary change in the face of adaptive challenges. In our sample, holding steady appears to have been aided by frequent exhibitions of partnering as public health officials created a thriving community among themselves. These officials found strength by exchanging support with peers and allies when faced with harassment. Additional qualitative research is needed to better understand these stories and to develop resilience training for public health officials to help them exercise these positive leadership behaviors during future crises.

The concept of work avoidance provides a useful lens for understanding the hostility directed towards public health officials [17]. The ALF offers an interpretation of harassment from the public not as the bad behavior of individual perpetrators or as failures in leadership; rather, harassment can be seen as an expected manifestation of adaptive resistance from people who feel their behaviors, beliefs, or values are being challenged by public health guidelines. These actions may reflect efforts to avoid the difficult adaptive work of questioning their own responsibility in the pandemic by blaming and attacking public health officials. With top-down harassment, the pattern in our data of public health officials being scapegoated and fired for promoting unpopular pandemic restrictions may reflect work avoidance by elected officials who redirected frustration towards public health officials rather than engage in the adaptive work of holding steady in the face of public criticism. We did not include work avoidance as a separate code in our analysis because we considered many reported incidents of harassment as examples of work avoidance within the ALF.

Our sample contained a few examples of “fighting back” after officials were fired or forced to resign from their positions of authority. For example, Rebekah Jones in Florida created her own data tracking site after losing her job as chief coronavirus data officer, and Michelle Fiscus, former medical director of Tennessee’s vaccine program, sued the state and spoke out regularly in national media outlets about being fired for conducting vaccine outreach to adolescents. These examples highlight how former officials exercised leadership without authority even after losing their official positions. In another case, the firing of Spokane Regional Health District health officer Bob Lutz led community members to form The Public Health Action Coalition Team of Spokane to hold the health board accountable. In this example, Lutz’s dismissal mobilized community members to fight for public health, demonstrating how acts of leadership can continue to pay dividends even when the authority figure loses power.

One ALF behavior that was underutilized in our sample was “giving work back”. For example, while our data revealed many instances of public health officials asking the public to comply with public health guidelines, they rarely offered ways for community members to contribute their perspectives to policy or strategy development. In a similar vein, while public health officials effectively partnered with other public health agencies, our data provided no examples of efforts to partner with organizations that might be opposed or suspicious of their work. Finding opportunities to share responsibility for solving adaptive problems with the public and other diverse stakeholders is a critical component of adaptive leadership [17]. As the world grows increasingly polarized, it will be essential for public health officials to increase their capacity for extending partnership opportunities to those who disagree with their approach. The scarcity of these examples in our data is consistent with the observation that giving work back (rather than offering top-down solutions) is not the traditional leadership approach for which authority figures are rewarded. It may also be that these cases are not perceived as being as newsworthy as other more dramatic events, as discussed below.

This study has several limitations. Because newspaper articles reflect editorial decisions and media framing processes, our sample is best interpreted as media portrayals of leadership during the pandemic rather than direct observations of all harassment incidents and leadership behaviors [23]. Nevertheless, examining these portrayals is valuable given the central role the media plays in shaping public understanding of public health crises and leadership [24]. Since news media are more likely to identify and publish high-profile or otherwise newsworthy cases, these types of stories are likely overrepresented in our sample of articles [25]. Thus, certain types of more newsworthy leadership behaviors (e.g., fighting back) may be more common in our data, and the leadership insights observed in these articles may be more transferable to higher-profile or more extreme incidents of harassment. An additional limitation relates to our search strategy in that, while EBSCO is a well-recognized database with broad national and regional coverage, it may not have captured all relevant media coverage. Since this analysis was limited to U.S. newspaper articles, the results may not generalize to other countries or media formats. The study also did not include additional efforts beyond reviewing the newspaper articles to attempt to verify or contextualize reported incidents. Finally, while we made every effort to ensure consistency in coding, formal intercoder reliability statistics were not calculated because all articles were jointly reviewed by both reviewers to reach consensus in the final stage of the coding process.

Although this study focuses on the U.S., similar reports of harassment toward public health professionals have emerged from around the world [9, 11, 26–30]. Our findings underscore the global importance of institutional protections, adaptive leadership capacity building, and long-term investments in recruitment and resilience for the public health workforce [31, 32]. Preparing now for future crises through deliberate workforce strengthening strategies will create more resilient systems better equipped to handle the public resistance that inevitably accompanies adaptive challenges.
