# Supplementary material for: Threats and harassment of public health officials in the United States during the COVID-19 pandemic: insights from the adaptive leadership framework
Source: Int J Public Health. 2026 Sep 1;71:1609739. doi: 10.3389/ijph.2026.1609739 (PMC13575384; doi:10.3389/ijph.2026.1609739)
Supplement: Supplementary file 1 [file Table1.docx]

**Supplementary File 1 – Distribution of Included Newspaper Articles by Publication Month (United States of America, 2026)**
